# *In Vitro* Flower Induction from Shoots Regenerated from Cultured Axillary Buds of Endangered Medicinal Herb *Swertia chirayita* H. Karst.

**DOI:** 10.1155/2014/264690

**Published:** 2014-02-25

**Authors:** Vikas Sharma, Barkha Kamal, Nidhi Srivastava, Anoop Kumar Dobriyal, Vikash Singh Jadon

**Affiliations:** ^1^Department of Biotechnology, Arni University, Kathgarh, Indora, Himachal Pradesh, India; ^2^Plant Molecular Biology Lab, Department of Biotechnology, Sardar Bhagwan Singh Post Graduate Institute of Biomedical Sciences and Research, Balawala, Dehradun, India; ^3^HNB Garhwal Central University, Campus Pauri, Pauri Garhwal, India

## Abstract

*In vitro* flowering and effective micropropagation protocol were studied in *Swertia chirayita*, an important medicinal plant using axillary bud explants. The Murashige and Skoog's medium (MS) supplemented with benzyl amino purine (BAP) 1.0 mg L^−1^ and adenine sulfate 70.0 mg L^−1^ was found optimum for production of multiple shoots. In the present study, incubation of flowering cultures on BAP supplemented medium (during shoot multiplication) was found necessary for flowering (6 weeks). However, concentrations of auxins-like IBA (0–2.0 mg/L) were ineffective to form reproductive buds. Subculture duration, photoperiod, and carbon source type do have influence on the *in vitro* flowering. The mature purple flowers were observed when the cultures were maintained in the same medium. This is the very first report that describes *in vitro* flowering system to overcome problems associated with flower growth and development as well as lay foundation for fruit and seed production *in vitro* in *Swertia chirayita*.

## 1. Introduction 

India is ranked the 6th among 12 mega diversity countries of the world [1] and Uttarakhand is one of the states in India which is known for its great diversity. *Swertia chirayita is *an important medicinal plantfound in Uttarakhand. *Swertia chirayita *is considered the most important plant for its bitterness, antihelminthic [2], hypoglycemic, hepatoprotective [3], and antiviral [4] properties. The novel techniques of plant tissue culture provide a viable alternative for managing these valuable resources in a sustainable manner. There are few reports about the tissue culture of *Swertia chirayita*. Documented literature reveals that there is a limited literature reported by few workers on *in vitro *propagation of *Swertia chirayita*, where they have used the nodal explants, *in vitro *grown seedlings, nodal meristems, and immature seed culture [5–9]. Balaraju et al. (2009) published reports on *in vitro *propagation of *S. chirata* using shoot tip explants derived from *in vitro *grown seedlings [10]. Chaudhuri et al. (2008) and Wang et al. (2009) reported direct shoot regeneration from *in vitro *leaves [11, 12]. But there is no report about the *in vitro* flowering of *Swertia chirayita *till date. The flowering process is one of the critical events in the life of a plant. This process involves the switch from vegetative stage to reproductive stage of growth and is believed to be regulated by both internal and external factors. A flowering system *in vitro* is considered to be a convenient tool to study specific aspects of flowering, floral initiation, floral organ development, and floral senescence [13]. The application of cytokinins, sucrose concentrations, photoperiod, and subculture time to promote flowering *in vitro* is well documented in many plant species [14, 15]. This is the very first report on *in vitro* flowering of this valuable medicinal plant and may open up new gates in the field of its conservation and continuous supply of plant material throughout the year by knowing its flowering behavior *in vitro. *This study is part of a larger programme designed to investigate the *in vitro *conservation protocol of *Swertia chirayita* and describes *in vitro* flowering system to overcome problems associated with flower growth and development as well as fruit and seed production *in vitro *and hence may open up new gates in the conservation and sustainable exploitation of this very important plant.

## 2. Materials and Methods

### 2.1. Plant Material

The nodal segments from juvenile plants of *Swertia chirayita* grown *ex situ* were collected from Hitech Nursery, Deovan, Chakrata (7,699 ft., lat. 30°43.642′, long. 77°51.941′), India, during the month of July and prepared herbarium was submitted to Botanical Survey of India, Northern Regional Centre, Dehradun (BSD), for identification of species level and plants were identified as *Swertia chirayita *(Roxb. ex Fleming) (VS 02) Family: Gentianaceae (Acc. number 113342). Surface sterilization was done as per the protocol given by Sharma et al. (2013) [16].

### 2.2. Culture Conditions

The basal media comprised of the mineral salts and organic nutrients of the MS medium (Murashige and Skoog, 1962) [17] containing 2.5% sucrose, solidified with 0.2% clarigel (HiMedia), and supplemented with 1.0 mg/L 6-benzylaminopurine (BAP) and 70 mg/L adenine sulfate was used for culture establishment [16, 18]. The Subculturing was performed at an interval of 3 to 4 weeks. Each treatment was replicated 12 times and all experiments were repeated at least thrice.

To examine the effect of photoperiod, 3 light/dark cycles, that is, 12/12, 16/8, and 8/16, were used in monitoring flowering *in vitro*. To examine the subculture time, explants were subcultured to fresh MS medium supplemented with 1.0 mg/L 6-benzylaminopurine (BAP) and 70 mg/L adenine sulfate on an interval of 4, 6, and 8 weeks. Five different sources of carbohydrates, that is, glucose sucrose, maltose, fructose, and lactose for a same concentration (2.5%), were examined for best flowering response. After bud formation, the cultures were shifted to continuous light of low intensity for induction of fully opened flowers. Subsequently, they were maintained under 16/8 h light/dark cycle for fruit development.

## 3. Results

**Figure 1:**
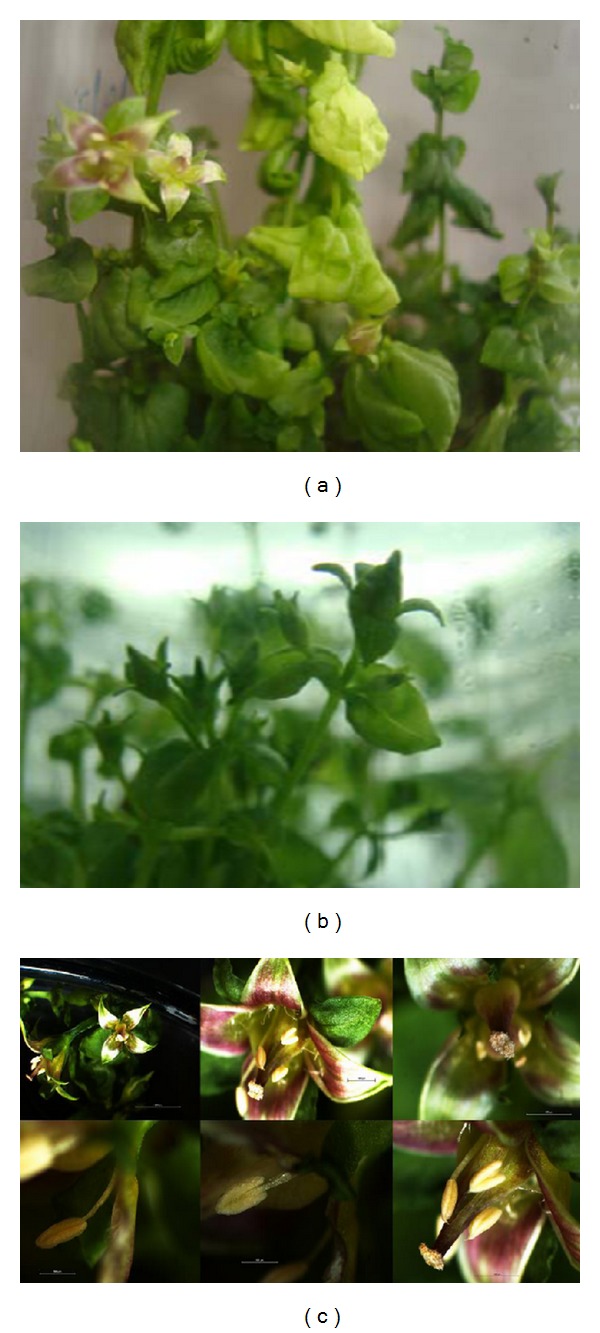
(a) *In vitro* flowering in *Swertia chirayita*. (b) Flower buds production. (c) Micrograph of *in vitro* flowers showing various parts of the flower and anthesis.

The *in vitro *flowering was observed in the present study of *Swertia chirayita *and had not been reported earlier. *In vitro *flowering offers a unique systemin the study of molecular basis and hormonal regulation of flowering. Flower initiated in MS media supplemented with BAP (1.0 mg/L) and adenine sulfate (70 mg/L) (Figure 1(a)) after 4–6 weeks of cultures. The production of flowering shoots continued for many subcultures spanning a period of more than two years. Flowers produced from tissue cultures systems presented normal morphological aspects. They were monoecious and differentiated from lateral branches as field-grown plants (Figure 1(c)). Besides, anthesis was observed in floral buds development.

Maximum numbers of flowers (buds) (12 per culture) were obtained when shoots werecultured on MS medium containing 1.0 mg/L BAP + 70 mg/L adenine sulfate (Figure 1(b)) and incubated at 16/8 h light/dark period after 6 weeks. In the present study, incubation of flowering cultures on BAP supplemented medium (during shoot multiplication) was necessary for flowering (6 weeks). However, concentrations of auxins-like IBA (0–2.0 mg/L) were ineffective to form reproductive buds (data not shown). The production of flowers was promoted in approximately the same proportion. Flowering was induced *in vitro* in excised shoot cultures of* Swertia chirayita *devoid of any preformed bud. Photoperiod was found to be important for *in vitro *flowering. Maximum *in vitro* flowers were obtained at 16 hrs ± 2 light periods; it was observed that plants incubated under 12 hrs or shorter photoperiods (8 hrs) were negatively affected for floral bud development. Optimum temperature for efficient *in vitro *flowering was 24°C ± 2°C with a relative humidity of 60–70%. The nature of carbon source (mono or disaccharides) in the medium has an important influence on the formation of reproductive buds. The carbohydrates slightly differed in their ability to support the formation of reproductive buds. In general, sucrose was best closely followed by glucose; maltose and fructose were also effective for formation of flowering shoots whereas lactose was totally ineffective (Table 1).

## 4. Discussion

Flowering is considered to be a complex process regulated by both internal and external factors and its induction under *in vitro *culture is extensively rare. Physiological studies have sought for many years about what is florigen and have shown that flowering time control is influenced by environmental factors and endogenous cues. Plants can integrate these signals, such as day length, vernalization, ambient temperature, irradiance, water/mineral availability, and presence/absence of neighbors, to relate flowering time. Flowering *in vitro *has been promoted by cytokinins at optimum concentrations.

Results obtained from our previous study [16] revealed that, after 4 weeks of initial culture, nodal explants cultured on MS medium with BAP (1.0 mg/L) and .007% (70 mg/L) adenine sulfate developed maximum number of multiple shoots and cytokinin especially that BAP with adenine sulfate was found to be the key component for multiple shoot establishment. In the present investigation, effectiveness of BAP in inducing bud break was observed and has been reported in many other plant species [19–22]. Cytokinin is a common requirement for *in vitro* flowering [23]. A number of studies report the use of cytokinins for *in vitro *flowering in species like *Murraya paniculata *[24], *Fortunella hindsii *[25], *Gentiana triflora *[26], *Pharbitis nil *[27] and *Ammi majus *[28]. There are reports that indicate the beneficial effects of cytokinin especially BAP on the induction of *in vitro *flowering for medicinal plants like *Withania somnifera, Rauvolfia tetraphylla, and Anethum graveolens *[29–31] which are in accordance with our investigation. BAP is found to be playing an important role not only as a growth regulator but also as a factor regulating floral organ formation of regenerated plantlets [32]. It has been reported that phytohormones affected flowering by mediating growth changes within the apical meristem and that cytokinins, in particular, played a key role in the initiation of mitosis and the regulation of cell division and organ formation. Auxins have frequently been reported to inhibit the formation of flowering buds *in vitro* in both long-day plants and short-day plants; low concentrations, however, may promote flowering even when higher ones are inhibitory [33]. Chrungoo and Farooq (1984) reported that, in saffron plants, NAA had an inhibitory effect on sprouting, vegetative growth, and flowering [34] and this has been in accordance with the present study where incorporation of IBA was not having promontory or inductive effect on flowering initiation. Carbohydrate source is also found to be an important factor and, in the present study, a lower concentration of 2.5% was found optimum for *Swertia chirayita* for flower initiation and maturation. This has been evident from study on* Arabidopsis thaliana *which reported that presence of sucrose in aerial parts of the plant promotes flowering [35]. Sucrose and cytokinins interact with each other for floral induction in *Sinapis alba *by moving between shoot and root.

Light is the most important environmental factor that induces changes in plant physiology and morphology, regulating flowering season cycles [36–38]. Day length and light quality play a crucial role in flower induction both *in vivo *and *in vitro *possibly due to altered photosynthetic turnover on flowering and are believed to be essentially perceived by expanded leaves; then, “florigen” (sucrose and isopentenyladenine) will be produced and moved directly or indirectly to shoot apical meristem (SAM) to guide flowering determination [39]. In some plants, vernalization alone is sufficient for flowering evocation, but others require subsequent exposure to inductive photoperiods (usually long days), and in them the changes at the apex wrought by vernalization and the photoperiodic stimulus are presumably different, possibly complementary [33]. *Swertia chirayita* is a high altitudinal plant enjoying gloomy and cold situation in nature, but importance of photoperiod instead of the vernalization for *in vitro *flowering of this plant has been demonstrated in the present study and maximum flowering frequency was observed with 16 h photoperiod. Subculture duration was also found to be an important factor for *in vitro* flowering in *Swertia chirayita* and importance of subculture duration has also been demonstrated by Wang et al. (2002) in other plant species [14].

## 5. Conclusion

In conclusion, our work has laid a preliminary foundation for a further research of *in vitro* flowering of *Swertia chirayita*. In tissue culture, *in vitro *flowering serves as an important tool for many reasons. One of the most important ones is being able to shorten the life cycles of plants; other aims include studying flower induction and initiation and floral development. Controlling the environment and media components enables the manipulation of different variables that affect these processes. So, this technique is of practical importance and can also serve for mass production of specific organs with unique compounds for pharmaceutical, nutritional, and other uses.

## Figures and Tables

**Table 1:** 

S. number	Photoperiod (light/dark)	Carbohydrate source					Subculture duration		
		S	G	F	M	L	4 wks	6 wks	8 wks
1	12 h/12 h	b	c	c	c	d	d	c	c
2	16 h/8 h	a	b	b	b	d	d	a	b
3	8 h/16 h	d	d	d	d	d	d	d	d

S: sucrose, G: glucose, F: fructose, M: maltose, and L: lactose (All 2.5%). Flowering response: a: average of 12 flower buds per culture, b: average of 6 flower buds per culture, c: average of 4 flower buds per culture, and d: no flowering. All cultures were maintained on full strength MS medium with 1 mg/L BAP and .007% adenine sulfate.
